# Standardization of Disinfectants

**Published:** 1913-07

**Authors:** 


Standardization of Disinfectants.—At Their Old Tricks.—The great "Council of Pharmacy"—Simmons' *instrument of persecution,* according to *American Medicine* has discriminated, in the very important matter of the standardization of disinfectants in favor of the test used in the Hygienic Laboratory as against that known as the Readal-Walker Test, in face of proof that the latter has answered every objection, and is almost universally adopted, and especially is officially adopted and used by the U. S. Insecticide Board of the Agricultural Department.

*American Medicine* says (May, 1913, p. 272): "For the Council of Pharmacy and Chemistry of the American Medical Association * * * from the ostensibly unbiased nature of their investigations, to reproduce 'objections' that have been proven incorrect and based on false premises, indicates either a woeful lack of knowledge of the subject they are discussing or very bad faith."

Further along, warming up as his indignation grows, Editor Lewis says (*loc. cit.*): "Such a spirit of unfairness and prejudice is incompatible, not alone with scientific research, but also with the ordinary instincts of honor and fair play." [Did you expect either, Dr. Lewis?]

---

Is a Doctor in Good Standing who repudiates and refuses to pay a small subscription bill, or any other honest debt? It is almost incredible, but it is nevertheless true, that there are amongst the members of the State Medical Association—members of county medical societies—men, some of whom are prominent and well known and well-to-do physicians, who have taken the "Red Back" year after year, who totally ignore my little polite reminders and utterly defeat every effort at collection, even through a Chicago collecting agency. Some of them owe me from $5 to $15, and they are deaf and dead to every kind of appeal. Some of these fellows were here at the big meeting, some delegates, perhaps, or holding some office. I am awfully tempted to give some of their names. Sometimes an insurance company asks me about the Texas doctors who apply for the appointment of examiner. One of the usual questions is: "Does he pay his debts?" Of course, I have long since ceased sending the JOURNAL to all who were in arrears five years; but there are many to whom I still send it who owe two to four years, and to whom bills are sent four times a year, without eliciting any reply. It is an awful bore and labor and expense to make out and mail and pay postage on these bills every three months. In many instances, it is neglect; but it is not excusable; but, in many other instances, it is pure cussedness and deadbeatism. Every fellow who reads this will know if the cap fits him. The great mass of my readers are, however, as true as steel, and ante, regularly, every year. May they live long and prosper. "Please remit." "Be good." "Do it now."

